# Diffusiophoresis of a Conducting Liquid Metal Droplet (LMD) in a Cylindrical Pore

**DOI:** 10.3390/molecules30163372

**Published:** 2025-08-13

**Authors:** Sunny Chen, Lily Chuang, Nemo Chang, Jean Chien, Venesa Liao, Eric Lee

**Affiliations:** Department of Chemical Engineering, National Taiwan University, Taipei 10617, Taiwan; f11524147@ntu.edu.tw (S.C.); f11524099@ntu.edu.tw (L.C.); f12524093@ntu.edu.tw (N.C.); f12524060@ntu.edu.tw (J.C.); r13524010@ntu.edu.tw (V.L.)

**Keywords:** diffusiophoresis, liquid metal droplet, LMD, conducting droplet, cylindrical pore, boundary confinement effect, double layer polarization, stagnation phenomenon, solidification phenomenon, drug delivery

## Abstract

Diffusiophoresis of a liquid metal droplet (LMD) in a cylindrical pore is investigated theoretically in this study. A patched pseudo-spectral method based on Chebyshev polynomials combined with a geometric mapping technique is adopted to solve the resulting governing electrokinetic equations in irregular geometries. Several interesting phenomena are found which provide useful guidelines in practical applications involving liquid metal droplets (LMDs) such as drug delivery. In particular, the severe boundary confinement effect brings about unique features of droplet motion, leading to mobility reversal and a “stagnation phenomenon” where droplets cease to move regardless of their surface charge densities in a narrow cylindrical pore. An overwhelming exterior vortex flow nearly enclosing the entire droplet is found to be responsible for this. This finds various practical applications in droplet microfluidics and drug delivery. For instance, a cylindrical pore or blood vessel may be clogged by a droplet much smaller than its radius. In addition, the “solidification phenomenon”, where all droplets move with identical speed regardless of their viscosities like rigid particles with no interior recirculating vortex flows, is also discovered. The electrokinetic mechanism behind it and its potential applications are discussed. Overall, the geometric configuration considered here is a classic one, with many other possible applications yet to be found by experimental researchers and engineers in the field of colloid industry and operations.

## 1. Introduction

Diffusiophoresis refers to the motion of a colloidal entity in response to a solute concentration gradient in a colloidal suspension. Compared with the well-known electrophoresis driven by an externally applied electric field, diffusiophoresis has no or negligible Joule heating effect, which makes it extremely attractive in biomedical applications like drug delivery, as an increase of four degrees Celsius is fatal to human cells and mammalian cells in general [1]. The self-guiding nature to migrate the colloidal entity following the concentration gradient established in the vicinity of the human tissue needing therapy enhances the concentration level of nanomedicines significantly; therefore, the overall therapeutic performance is maximized there [2], as specific chemicals are often released into the bodily fluid in the infected region. These concentration gradients not only play the role of directional guidance but also serve as the driving force for drug-loaded nanomedicines [3,4,5]. This is often achieved with liposomes, where therapeutic ingredients are encapsulated in a liquid droplet enclosed by a lipid bilayer. In addition, liquid droplets in general have many merits which enable them to also be very attractive in various other practical applications. For instance, as a colloidal entity, the tiny droplet is an almost-ideal chemical reactor characterized by fast thermal transfer, efficient mixing, and a narrow residence time [6]. In particular, coupled with diffusiophoresis, droplets have also found significant applications in enhanced oil recovery (EOR), where seawater is pumped into the porous oil reservoir to drive out the oil droplets contained in the void space with the driving force provided by the diffusiophoresis [7].

As mentioned above, diffusiophoresis of droplets has also found considerable applicability in biomedical applications such as drug delivery. Beyond the conventional liposome droplet introduced above, which is filled with dielectric fluids in general, a new class of materials has recently emerged as well in the biomedical field in the form of liquid metal droplets (LMDs) [8,9,10]. Liquid metals refer to metals that are in the form of a liquid at room temperature [11,12], such as the well-known mercury. Thanks to their softness and advantageous physicochemical traits—such as biocompatibility, flexibility, high electrical conductivity, and surface modifiability—liquid metals stand out as promising candidates for next-generation biomaterials [11,13]. These properties make them ideal for applications requiring stable, conductive droplets that remain liquid at physiological temperatures. In microfluidics, on the other hand, their high thermal conductivity (29.3 W/m·K for gallium [11]) also allows LMDs to serve as cooling agents, effectively removing heat from hotspots via conduction and convection [14]. Their high electrical conductivity and mechanical stability further expand their utility, enabling them to function as efficient microscale pumps [15] or to drive chaotic internal mixing [16], thereby significantly enhancing fluid transport and mixing capabilities in microfluidic systems.

Among them, gallium-based LMDs, including EGaIn (75% Ga, 25% In by weight) and galinstan (68.5% Ga, 21.5% In, 10% Sn), have gained particular attention in both microfluidics and drug delivery. This is largely due to their low toxicity, low vapor pressure, and low melting points (Ga: 29.8 °C, EGaIn: 15 °C, galinstan: −19 °C), which allow them to remain in a liquid state under physiological conditions [11,12]. Gallium and its alloys have been found to have significant therapeutic functions in biomedical applications. Although the precise mechanism is not clear yet, the similarity of many of their chemical properties to those of iron makes it possible for them to bind to iron-containing proteins, such as the iron transport protein transferrin [17]. Consequently, microorganisms may mistakenly utilize gallium instead of iron for essential iron-dependent cellular activities that support viability and growth, such as the transportation of oxygen. This interference has been explored for its therapeutic potential in various diseases, as highlighted in recent reviews [18,19]. Consequently, LMDs of gallium and their alloys become nanomedicines themselves in drug delivery operations, with no encapsulation into the drug carrier needed, as required in conventional liposomes. Moreover, the LMD surface can be grafted with specific chemical compounds or polymers to modify its physical or pharmaceutical properties as desired, such as changing its polarity or forming a concentric outer porous layer [20,21]. As their alloys can be fabricated essentially at will, this opens the gate to a wide range of possible new materials with designed desirable properties in their practical applications. Indeed, this field has become a promising new research field full of innovations and possibilities.

Diffusiophoresis of droplets, either dielectric or conducting, in an infinite medium of electrolyte solutions, either dielectric or conducting, has been investigated extensively in recent years by Lee and his coworkers [3,4,22,23], motivated by its potential applications mentioned above, such as in microfluidic operations and drug delivery. Note that it is presumed that there is no nearby boundary present so that the system can be regarded as an infinite medium. However, such a boundary is often present in many practical applications. Corresponding studies for the diffusiophoresis of conducting droplets in a confined geometry, such as a cylindrical pore, however, have not been conducted yet, to the best of our knowledge. This is a very important issue as the presence of a nearby solid wall will have profound boundary confinement effect upon the droplet motion, both hydrodynamically and electrostatically, owing to the severe steric deformation of the electric double layer surrounding the droplet by the solid boundary, which has a significant impact upon the droplet electrokinetic motion in general. This is particularly important for the application in microfluidics, as a cylindrical pore is frequently encountered in the form of a capillary, the fundamental unit in microfluidic operations. Moreover, capillaries of the size 200 nanometers in radius are commercially available now, and a size as small as 5 nanometers can be fabricated in the lab as well. As a result, the droplets within such narrow capillaries often migrate under the influence of a nearby solid wall of the cylindrical pore. The boundary confinement effect cannot be overlooked anymore in this case. It is a fundamental classic issue to be reckoned with in electrokinetics after all, which is by far one of the very challenging ones due to the irregular geometry of the boundary involved. In addition, the blood vessels encountered in drug delivery are often regarded and treated as a cylindrical pore as well. Obviously, diffusiophoresis of a conducting droplet in a cylindrical pore also has direct application there.

With the motivation elaborated as above on the importance both in terms of the fundamental understanding of a classic electrokinetic problem and its huge potential in practical applications like microfluidic operations and drug delivery, we propose here to conduct a theoretical analysis on the diffusiophoresis of a conducting liquid metal droplet (LMD) of an arbitrarily charged level in a cylindrical pore, focusing on the boundary confinement effect of the nearby solid pore wall. A corresponding study for the diffusiophoresis of a dielectric droplet in a cylindrical pore has been reported very recently in the literature by Lee and his coworkers [5], where the Maxwell traction tangent to the droplet surface is present. However, for the conducting LMDs considered here, there is no such electrostatic force present tangent to the droplet surface. They are horses of different colors, electrokinetically speaking. Moreover, the dielectric droplet considered there is weakly charged, whereas here we extend it to highly or arbitrarily charged conducting liquid metal droplets to explore the impact of the droplet surface electric condition. The effects of electrokinetic interest are examined in detail, such as the surface charge density, the size of the cylindrical pore, and so on. The possible utilization of the results in practical applications is elaborated as well.

## 2. Results

Mobility profiles and flow fields of the droplet motion are obtained under various electrokinetic conditions with the numerical algorithm developed based on the patched pseudo-spectral method. Corresponding specific discussions for each and every such result are contained in the following Discussion section.

## 3. Discussion

**Figure 1:**
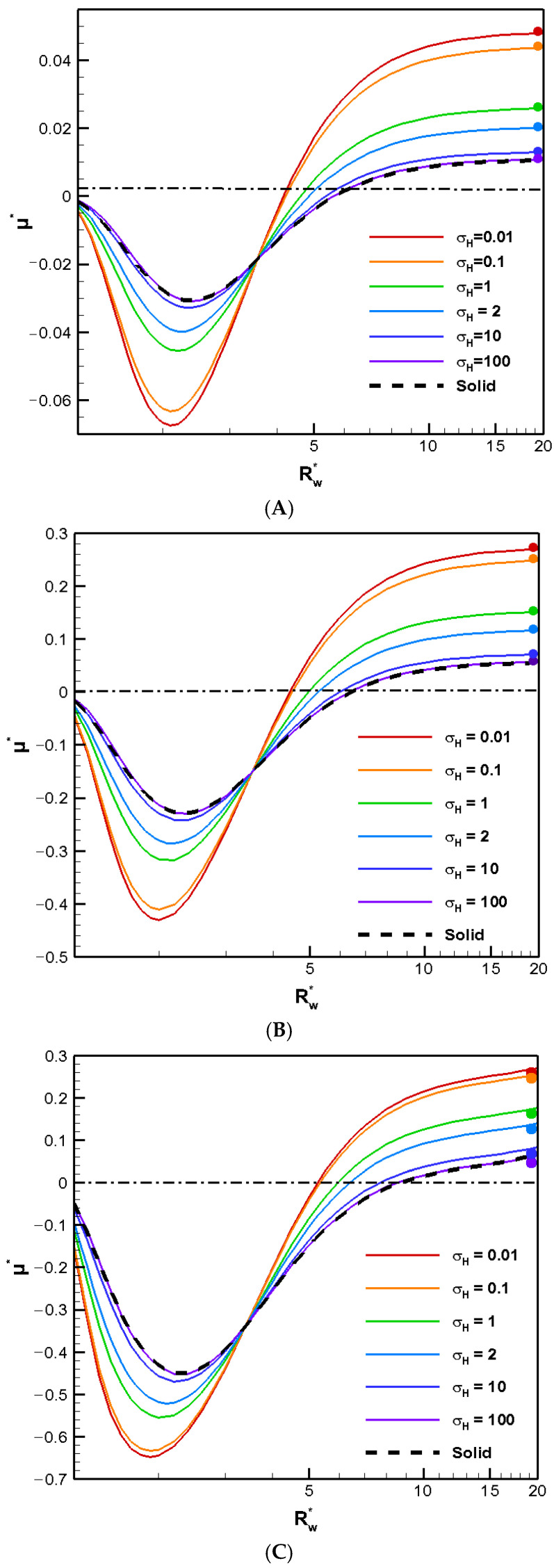
Dimensionless mobility (μ*) as a function of $R_{w}^{*}$ at various viscosity ratio σ_H_ for conducting droplets with κa = 1 in the KCl solution (β = 0). (**A**) σ* = 2.03; (**B**) σ* = 6.94; (**C**) σ* = 15.79.

The boundary confinement effect of a nearby cylindrical pore is demonstrated in Figure 1, where the diffusiophoretic mobility of a conducting droplet in general is depicted as a function of $R_{w}^{*}$, the ratio of the pore radius to the droplet radius, for various droplet viscosity ratios to the ambient solution. The surface charge levels are expressed based on the surface charge density, which ranges from a weakly charged one (σ* = 2.03) to highly charged one (σ* = 6.94) and an even higher one (σ* = 15.79) in Figure 1A, Figure 1B, and Figure 1C, respectively. The value of κa, the dimensionless reciprocal of the double layer thickness, which is a measurement of the double layer thickness, is set to unity. The larger the κa value is, the thinner the double layer is in general. For κa = 1, the characteristic double layer thickness is equal to the droplet radius. The actual double layer thickness, however, may be three to four folds of the droplet radius [24]. Note that this is the thickness of the double layer where the effect of the double layer polarization is most profound [25]. As a result, the corresponding motion-inducing effect in chemiphoresis is strongest when the double layer thickness is comparable to the droplet radius, i.e., $R_{w}^{*}$ around 2.0 here. This is exactly what we observe in Figure 1. When $R_{w}^{*}$ equals unity, the droplet is trapped within the cylindrical pore and hence ceases to move. Therefore, the droplet mobility reduces to zero, as shown in Figure 1. On the other hand, as shown in Figure 1, when $R_{w}^{*}$ reaches 20, the presence of the boundary is hardly felt, and the droplet mobilities of various viscosities reduce to their corresponding single droplet situations, indicated by the colored solid dots [4]. The less viscous the droplet is, the faster the droplet moves in general, as anticipated from the purely hydrodynamic consideration based on the modified Stokes law for droplets [24]. The absence of the upward Maxwell traction tangent to the conducting droplet surface significantly simplifies the flow field around the droplet. Note that the vertical component of the corresponding Maxwell traction normal to the droplet surface still yields an upward electric driving force, which is strong enough to overpower the downward hydrodynamic drag force resulting from the nearby electro-osmosis flow moving down. As a result, the droplets still move upward along the concentration gradient following the direction of the local electric field induced by the motion-inducing double layer polarization in chemiphoresis, i.e., β = 0 [25]. In other words, positive mobility is generally observed in an infinite medium of KCl solution regardless of the droplet viscosity, as shown in Figure 1 by the colored dots. Moreover, this tendency remains unchanged when the pore is not too close to the droplet. However, the droplet moves somewhat more slowly as the pore becomes narrower, owing to the motion-retarding hydrodynamic drag force which increases as the pore becomes narrower.

**Figure 2:**
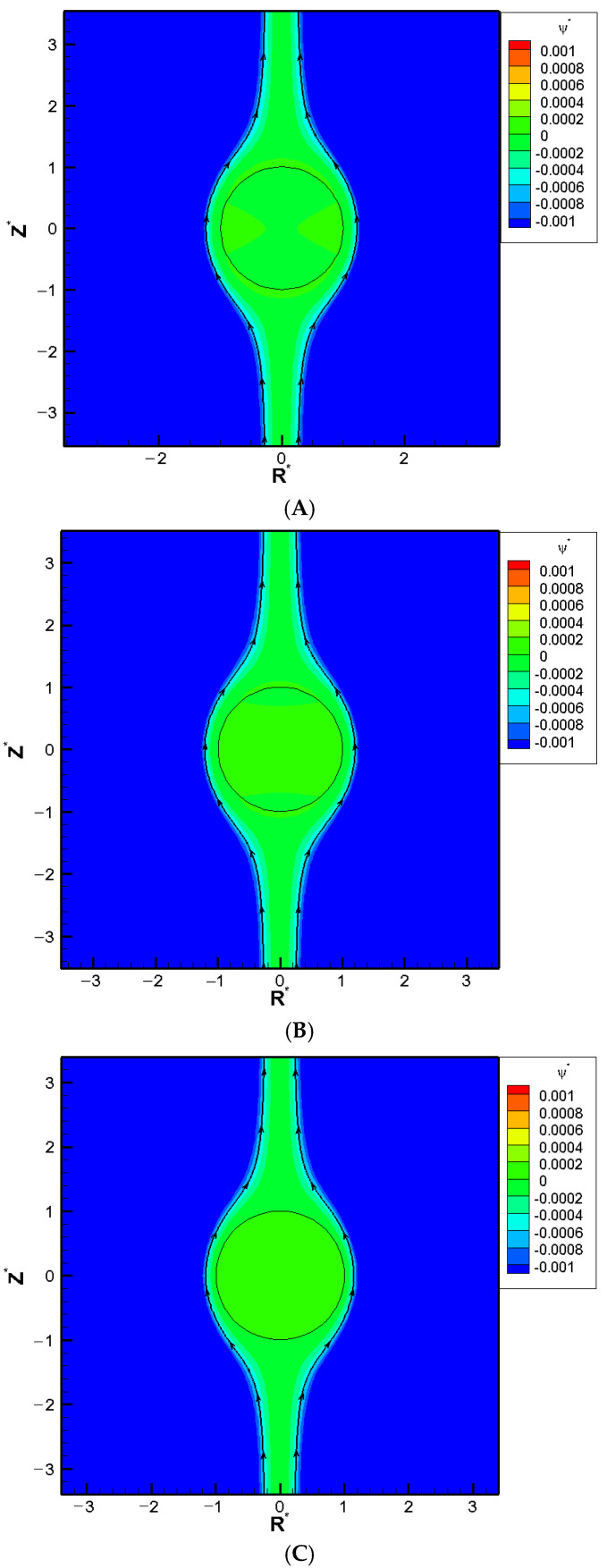
Contour plots of stream function for a conducting droplet with σ_H_ = 2 and κa = 1 in the KCl solution (β = 0), where R* is the coordinate in the radial direction and Z* is the coordinate in the longitudinal direction: (**A**) σ* = 2.03, $R_{w}^{*}$ = 3.55; (**B**) σ* = 6.94, $R_{w}^{*}$ = 3.52; (**C**) σ* = 15.79, $R_{w}^{*}$ = 3.40.

When the pore becomes even narrower, however, a severe boundary confinement effect starts to be revealed. Mobility reversal happens eventually, regardless of the droplet viscosity. The droplets now move downward instead. The more viscous the droplet is, the earlier this happens as the cylindrical pore becomes narrower. This is because the drastic reduction in the cross-sectional area for fluid to flow through around the droplet, which leads to a significant buildup of the fluid in the upstream region of the droplet. The hydrodynamic pressure increases there as a result and pushes the droplet downward instead. A negative droplet mobility is thus observed as the cylindrical pore becomes narrower. Moreover, an interesting phenomenon is observed where the droplet moves like a rigid particle with identical speed regardless of its viscosity and surface charge density, which is referred to as the “solidification phenomenon” here as a result. No recirculating interior vortex flow is present, as demonstrated in Figure 2A–C. This is because the two hydrodynamic spinning forces upon the droplet surface with opposite orientations reach a deadlock. These two spinning forces are generated by the downward diffusio-osmosis flow in the bulk electrolyte solution, and the upward flow surrounding the droplet moving downward. The surface shear rate is zero as a result, which makes the droplet resilient against possible external undesirable damaging shear stresses in its migration. This has significant importance for practical applications such as drug delivery. The specific κa value where the solidification phenomenon happens provides a guideline of the optimum liposome droplet size in the fabrication stage of nanomedicines. Liposomes with this size will have the strongest resilience against possible external damaging stress, as elaborated above. Corresponding flow fields at the solidification points where solidification phenomenon happens are shown in Figure 2A–C for different surface charge densities.

**Figure 3:**
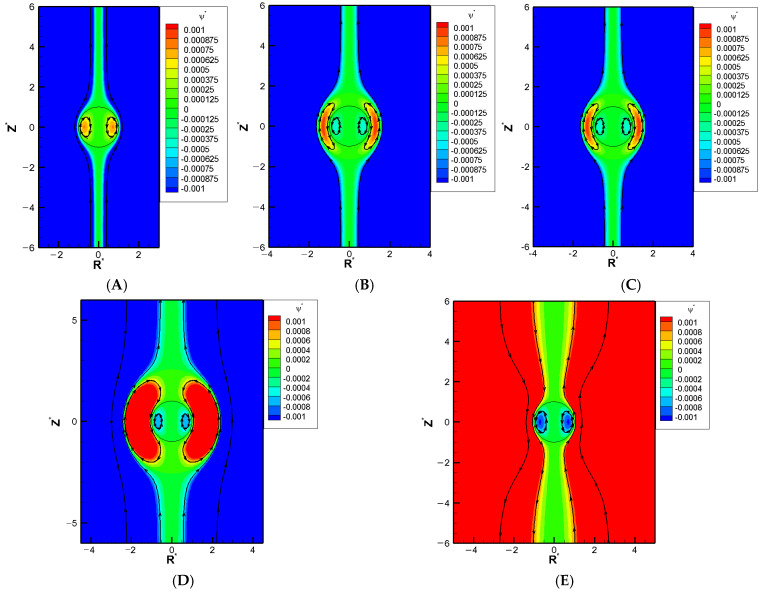
Contour plot of stream function with σ_H_ = 2.0, κa = 1, and σ* = 2.03 in the KCl solution (β = 0), where R* is the coordinate in the radial direction and Z* is the coordinate in the longitudinal direction: (**A**) $R_{w}^{*}$ = 3; (**B**) $R_{w}^{*}$ = 3.55; (**C**) $R_{w}^{*}$ = 4; (**D**) $R_{w}^{*}$ = 4.5; (**E**) $R_{w}^{*}$ = 5.

We show a series of flow fields of the galistan liquid metal droplet $(\sigma_{H}\;$= 2) in particular as a representative benchmark LMD with decreasing $R_{w}^{*}$., as shown in Figure 3A–E. An exterior vortex flow surrounding the droplet starts to appear in Figure 3B as the pore becomes narrow enough. A closer look shows that the orientation of this exterior vortex tends to spin the droplet downward, in other words, enhances the hydrodynamic drag force from the downward electro-osmosis flow. As a result, the overall downward pushing force becomes greater than the electric upward driving force from the Maxwell traction normal to the droplet surface. This leads to the mobility reversal eventually. This is consistent with the deduction we made in Figure 1 based on the buildup of hydrodynamic pressure.

**Figure 4:**
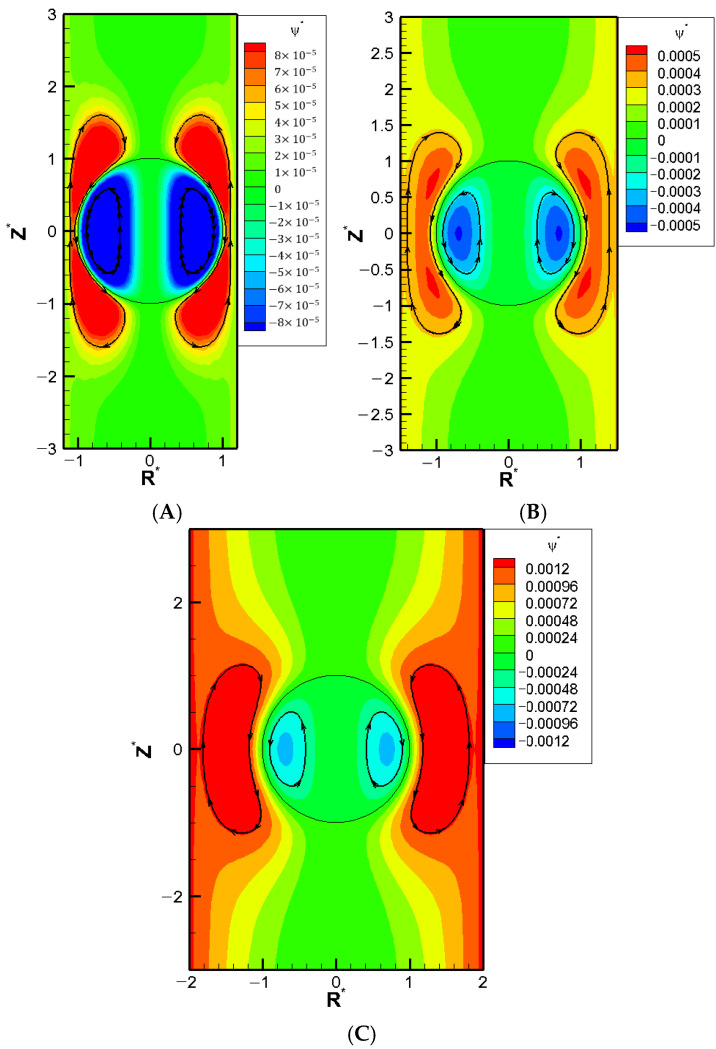
Contour plot of stream function with σ_H_ = 2.0, κa = 14.16, σ* = 2.03, and $R_{w}^{*}$ = 1.2 in the KCl solution (β = 0), where R* is the coordinate in the radial direction and Z* is the coordinate in the longitudinal direction: (**A**) $R_{w}^{*}$ = 1.2; (**B**) $R_{w}^{*}$ = 1.5; (**C**) $R_{w}^{*}$ = 2.0.

The boundary confinement effect from the narrow pore is equivalent to an enhancement effect over the double layer polarization, which is motion-inducing in chemiphoresis. As the double layer polarization is most significant when its thickness is comparable to the droplet radius, which transfers to $R_{w}^{*}$ around two, the largest downward droplet should happen for $R_{w}^{*}$ around two, which is exactly what is observed here in Figure 4. Beyond this point, the pore gets so narrow and the double layer becomes so severely squeezed that most of ions cannot pass through the droplet at ease. Severe backflow happens pushing back the electroosmosis flow as well. The droplet flow is slowed down as a result, leading to a decrease of droplet mobility. Finally, the droplet is virtually trapped in the cylindrical pore when $R_{w}^{*}$ is equal to unity and the droplet mobility becomes zero. This is exactly what is observed in Figure 4A, local minimum of each and every mobility profile in Figure 4 is observed for $R_{w}^{*}$ around two, which is exactly what it should be based on our discussion above: the boundary confinement effect affects the droplet motion via the squeezing effect upon the double layer.

**Figure 5:**
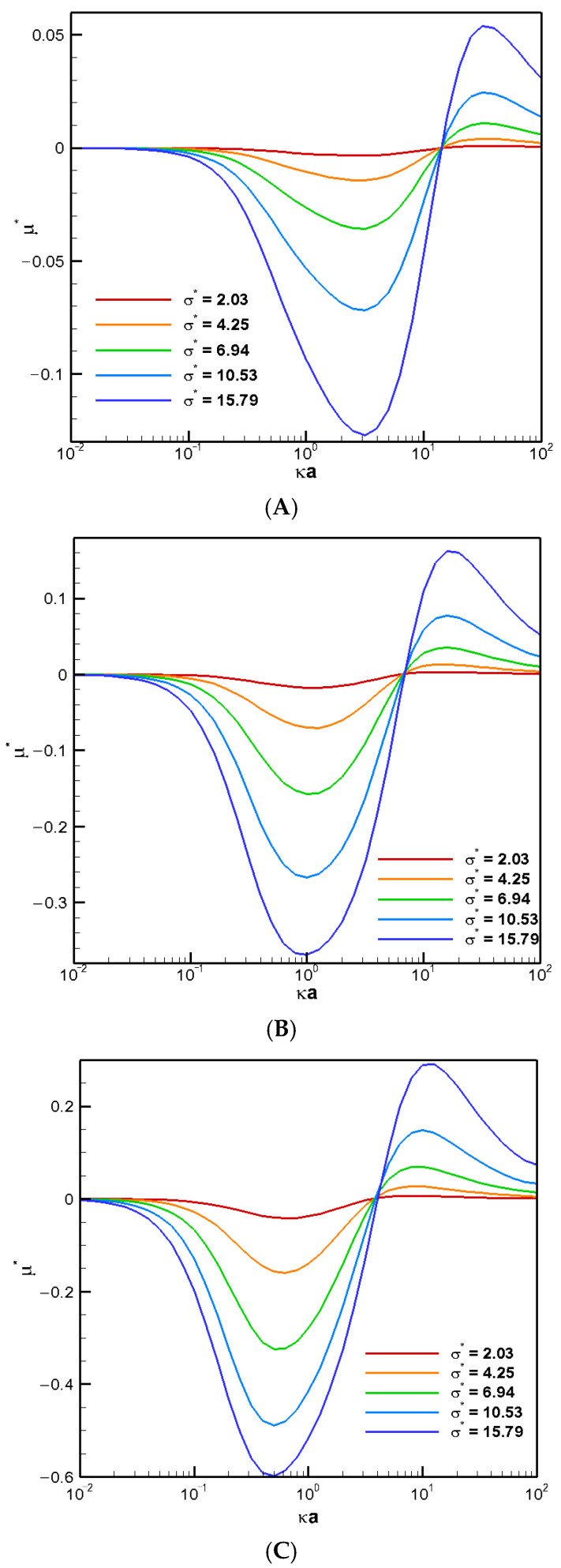
Dimensionless mobility as function of κa with various $\sigma^{*}$ for conducting droplets in a cylindrical pore with σ_H_ = 2.0 in the KCl solution (β = 0) (**A**) $R_{w}^{*}$ = 1.2; (**B**) $R_{w}^{*}$ = 1.5; (**C**) $R_{w}^{*}$ = 2.

We now go on to investigate the corresponding mobility profiles as the droplet surface charge density increases. As shown in Figure 5, a “stagnation point” where the droplet mobility becomes zero is observed regardless of the droplet surface charge level. The corresponding flow fields at these stagnation points are shown in Figure 4 for various surface charge densities. Large exterior vortex flows surrounding the droplet extend to both upstream and downstream regions, essentially enclosing the entire droplet. Hydrodynamically speaking, these strong recirculating vortex flows retard the downward bulk fluid flow passing the droplet. Instead, the fluid is forced to circulate around the droplet, forming large vortex flows that nearly enclose the entire droplet. As a result, the droplet becomes hydrodynamically trapped in its original place in a sense. Moreover, the electrostatic screening/sheltering effect resulted from the large exterior vortex flow that covers essentially the entire droplet surface might be the corresponding electrostatic mechanism behind it as well. The effective surface charge density reduces to essentially zero due to the offset by those closely present counterions in the double layer, which is driven so close to the droplet surface by these exterior vortex flows. The green color near the centerline indicates that these droplets are motionless with zero droplet mobilities. However, the recirculating vortex flows both inside and outside the droplet continue to move around. Similar stagnation phenomenon has also been reported experimentally in other systems [26].

The stagnation phenomenon highlights a potential problem in microfluidic operations. The entire channel may become clogged even when the droplet size is smaller than the cylindrical pore diameter. Notably, this phenomenon occurs regardless of the droplet’s surface charge levels, indicating that the hydrodynamic trapping due to the boundary confinement effect may be the ultimate electrokinetic mechanism underneath it after all. The stagnation phenomenon appears for cylindrical pores sufficiently narrow over a range of $R_{w}^{*}$, as shown in Figure 5A–C. The precise location of κa may be slightly different, but the overall behavior is quite similar.

## 4. Theory

**Figure 6:**
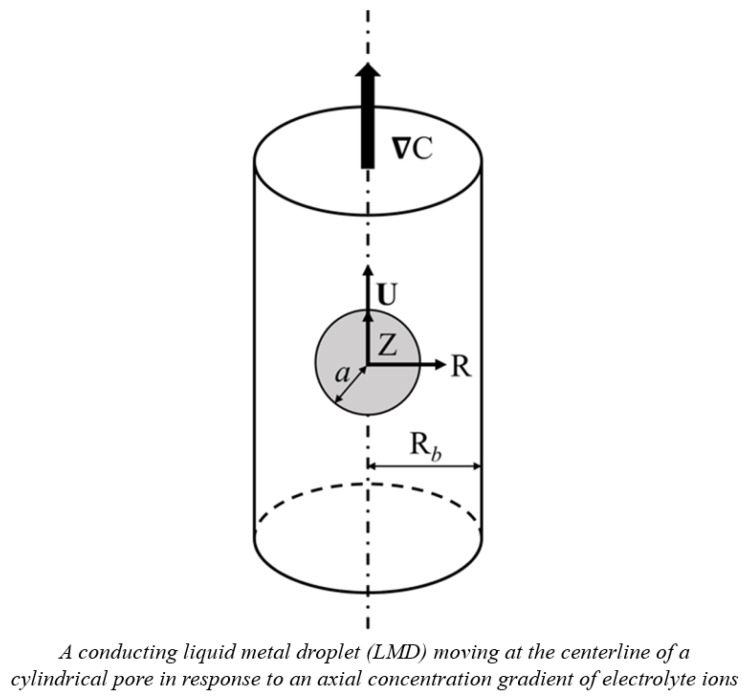
System diagram.

As shown in Figure 6, the diffusiophoretic motion of a charged, conducting liquid metal droplet (LMD) in a binary KCl electrolyte solution is considered here. A concentration gradient of ions, **∇**C, is applied in the Z-direction which drives the droplet in motion in the same direction. The electrolyte solutions considered are assumed to be Newtonian, incompressible, and have constant material constants like viscosities and dielectric constants [25]. The surface of the conducting droplet is assumed to be mobile, ion-impenetrable, and remain spherical without deformation while in motion, which is justified by the extremely low hydrodynamic Weber number, typically around 10^−7^ [4]. Furthermore, the droplet surface is assumed to be equipotential, which is true for conducting droplets due to their extremely high electrical conductivity. It is also true for droplets whose surface charges are solely from ion adsorption. All the charges are assumed to be uniformly distributed on the droplet surface, and the interior fluid contains no electrolyte ions. Moreover, the surface charge density is assumed to remain constant with varying electrokinetic environments characterized by the electrolyte strength in the ambient solution. In other words, these ions are indifferent ions which will not adsorb to the droplet surface regardless of their concentrations [22,27]. Based on these assumptions, the governing equations are as follows:


(1) $$\nabla^{2}\phi =-\frac{\rho }{\varepsilon_{m}}\;a\leq r<R_{b}$$

(2) $$\nabla^{2}\phi_{I}=-\frac{\rho }{\varepsilon_{D}}\;0\leq r<a$$

(3) $$f_{j}=-D_{j}\left(\nabla n_{j}+\frac{z_{j}e}{k_{B}T}n_{j}\nabla \phi \right)+n_{j}v\;a\leq r<R_{b}$$

(4) $$\nabla \cdot f_{j}=0\;a\leq r<R_{b}$$

(5) $$\eta_{m}\nabla^{2}v-\nabla P-\rho \nabla \phi =0\;a\leq r<R_{b}$$

(6) $${\eta_{D}\nabla }^{2}v_{I}-\nabla P_{I}=0\;0\leq r<a$$

(7) $$\nabla \cdot v=0\;a\leq r<R_{b}$$

(8) $$\nabla \cdot v_{I}=0\;0\leq r<a$$

Equation (1) is the Poisson equation with ϕ defined as the local electric potential, ρ the local space charge density, and ε_m_ the electric permittivity of the ambient electrolyte solution. Equation (3) is the famous Nernst–Planck equation governing the migration of ions with **v** as the local velocity of the fluid in the electrolyte solution exterior to the droplet. Equation (4) is based on the conservation of all ions at quasi-steady state. Equation (5) is the modified Stokes equation taking into account the electric body force due to the presence of ions in the ambient electrolyte solution, −ρ∇ϕ, where P is the local hydrodynamic pressure. Equation (6), on the other hand, is simply the classic Stokes equation with suffix I indicating the physical quantities in the interior droplet fluid. Equations (7) and (8) are simply the incompressibility constraints of the fluids outside and inside the droplet, respectively.

The problem is a two-dimensional one due to its axisymmetric nature, which eliminates any dependence on the azimuthal angle φ. Standard linear perturbation analysis is then adopted, assuming that the system is only slightly perturbed from the equilibrium state [26].

### 4.1. Boundary Conditions

Corresponding boundary conditions on the droplet surface are as follows:

(9) $${\varepsilon_{m}\left[\frac{\partial \phi_{e}}{\partial r}\right]}_{r=a^{+}}-\varepsilon_{D}{\left[\frac{\partial \phi_{eI}}{\partial r}\right]}_{r=a^{-}}=-\sigma \;r=a$$

(10) $$\frac{\partial \phi_{e}}{\partial r}=-\sigma \;r=a$$

(11) δϕ=0 r=a

(12) $$\frac{\partial g_{1}}{\partial r}=0\;r=a$$

(13) $$\frac{\partial g_{2}}{\partial r}=0\;r=a$$

(14) $${\left.\frac{\partial \Psi }{\partial r}\right|}_{r=a^{+}}={\left.\frac{\partial \Psi_{I}}{\partial r}\right|}_{r=a^{-}}\;r=a$$

(15) $$\tau_{r\theta }^{N}{|}_{r=a^{+}}=\tau_{r\theta I}^{N}{|}_{r=a^{-}}\;r=a$$

Equation (9) is derived from the two-dimensional Gauss divergence theorem, which reduces to Equation (10) in the case of a conducting droplet, where the internal potential gradient vanishes. In other words, there is no dependence on the droplet permittivity for a conducting droplet in general. This simplification occurs because the general Poisson equation reduces to the Laplace equation in the chargeless interior fluid [28], leading to a null internal potential gradient, thus eliminating the contribution from the droplet’s electric permittivity.

Here we focus on the conducting LMDs, which are normally regarded as ideally polarizable with constant surface charge density invariant of all the parameters in the system. The perturbation in electric potential, described in Equation (11), reflects the exceptionally high electrical conductivity of a conducting droplet. The impermeability of the droplet is implied by Equations (12) and (13). Equation (14) describes the continuity of the tangential fluid velocity at the droplet interface. Equation (15) corresponds to the classic Rybczynski–Hadamard condition, indicating that the hydrodynamic shear stress remains continuous across the droplet interface. Note that the superscript N here indicates the hydrodynamic stress. For a conducting droplet, only hydrodynamic viscous stress needs to be considered, as the surface is equipotential; hence, there is no extra involvement of the electric Maxwell traction tangent to the droplet surface [3,29].

For a non-conducting cylindrical pore with no-slip and non-permeable conditions on the pore surface, the boundary conditions there are given as follows:

(16) $$\phi_{e}=\phi_{w}=0\;r=R_{w}$$

(17) $$\frac{\partial \delta \phi }{\partial R}=0\;r=R_{w}$$

(18) $$\frac{\partial g_{j}}{\partial R}=0\;r=R_{w}$$

(19) $$\psi =0\;r=R_{w}$$

(20) $$\frac{\partial \psi }{\partial R}=0\;r=R_{w}$$

Far away from the droplet, the concentration and flow fields resemble those in a system without the droplet. In other words, fully developed conditions with no change in the Z-direction for each and every variable are adopted in general, except for the imposition of the concentration gradient there. Therefore, the equilibrium state of a cylindrical pore without the droplet is used to define the boundary conditions at infinity. The electric potential distribution in a chargeless pore, denoted as $\phi_{e,\infty }\left(R\right)$, is set to zero. The velocity field at infinity, $\psi_{\infty }\left(R\right)$, is also zero, as no diffusio-osmotic flow is generated along a chargeless cylindrical pore wall. Here, L represents a sufficiently large distance beyond which further increases in L no longer affect the droplet mobility.

(21) $$\psi =\frac{1}{2}UR^{2}+\psi_{\infty }\left(R\right),\frac{\partial \psi }{\partial Z}=0\;Z=\pm L$$

(22) $$\phi_{e}=\phi_{e,\infty }\left(R\right)=0\;Z=\pm L$$

(23) $$\frac{\partial \delta \phi }{\partial Z}=-\beta \;\nabla C\;Z=\pm L$$

(24) $$g_{j}=\left(\beta -1\right)\nabla C\;r\;cos\theta \;Z=\pm L$$

These dimensional equations are converted to corresponding dimensionless form by dividing each dimensional variable with a selected suitable characteristic variable together with the same non-dimensionlization procedure of the boundary conditions. The characteristic variables selected are set as follows:

The size of the spherical droplet is characterized by its radius denoted by a (m). The ambient electrolyte solution surrounding the droplet is characterized by an electric permittivity ε_m_, with unit as Coulomb per volt per meter (C/V/m). The (number) concentration of the system is characterized by the total concentration of electrolyte ions in the bulk solution, C_0_; meanwhile, the (number) concentration of each ionic species j is denoted as n_j0_, both with units as (1/m^3^). Moreover, as there is no available velocity in the system to serve as the natural choice, in order to non-dimensionalize the diffusiophoretic velocity of the droplet appropriately, we define an auxiliary characteristic velocity U_0_ as follows:

(25) $$U_{0}=\frac{\varepsilon_{m}}{\eta_{m}a}{\left(\frac{k_{B}T}{z_{j}e}\right)}^{2}$$

where η_m_ and ε_m_ are the viscosity and the electric permittivity of the electrolyte solution, respectively; k_B_ is the Boltzmann’s constant; T is the absolute temperature (K); z_j_ is the valence of the ionic species j; and e is the elementary charge. The resulting unit by this definition yields the expected velocity dimension as (m/s). With the introduction of these characteristic variables, the entire system of governing equations and the associated boundary conditions can be converted to dimensionless form based on the following non-dimensionalization procedure of involved variables as follows:

(26) $${R_{w}}^{*}\equiv \frac{R_{w}}{a},\;L^{*}\equiv \frac{L}{a},\;r^{*}\equiv \frac{r}{a}\;and\;R^{*}\equiv \frac{R}{a}\;,$$

(27) $$Z^{*}\equiv \frac{Z}{a},\;\phi_{e}^{*}\equiv \frac{\phi_{e}}{\phi_{0}},\;\delta \phi^{*}\equiv \frac{\delta \phi }{\phi_{0}}\;,$$

(28) $$g_{j}^{*}\equiv \frac{g_{j}}{\phi_{0}},\;n_{j}^{*}\equiv \frac{n_{j}}{n_{10}},\;{E_{ex}}^{*}\equiv \frac{E_{ex}}{\phi_{0}/a},$$

(29) $$\psi^{*}\equiv \frac{\psi }{U^{0}a^{2}},\;\sigma_{H}\equiv \frac{\eta_{D}}{\eta_{m}},\;U^{*}\equiv \frac{U}{U_{0}},$$

(30) $$\phi_{w}^{*}\equiv \frac{\zeta_{w}}{\phi_{0}},\;\sigma^{*}\equiv \frac{\sigma a}{\varepsilon_{m}\phi_{0}},\;\kappa^{-1}\equiv \sqrt{\sum_{j=1}^{N}\frac{\varepsilon_{m}k_{B}T}{{n_{j0}\left(z_{j}e\right)}^{2}}}$$

The resulting governing equations in dimensionless form are as follows.

(31) $$\nabla^{*2}\phi_{e}^{*}+\frac{{\left(\kappa a\right)}^{2}}{1+\alpha }[\exp\left(-\phi_{e}^{*}\right)-\exp\left({\alpha \phi }_{e}^{*}\right)]=0\;1\leq r^{*}<R_{w}^{*}$$

(32) $$\nabla^{*2}\phi_{e_{I}}^{*}=0\;0\leq r^{*}<1$$

(33) $${\nabla^{*}}^{2}\delta \phi^{*}-\frac{(\kappa a{)}^{2}}{1+\alpha }\left[\exp(-\phi_{e}^{*})+\alpha \exp(\alpha \phi_{e}^{*})\right]\delta \phi^{*}=\frac{(\kappa a{)}^{2}}{1+\alpha }\left[\exp(-\phi_{e}^{*})g_{1}^{*}+\alpha \exp(\alpha \phi_{e}^{*})g_{2}^{*}\right]\;1\leq r^{*}<R_{w}^{*}$$

(34) $$\nabla^{*2}\delta \phi_{I}^{*}=0\;0\leq r^{*}<1$$

(35) $${\nabla^{*}}^{2}g_{1}^{*}-(\frac{\partial \phi_{e}^{*}}{\partial r^{*}}\frac{\partial g_{1}^{*}}{\partial r^{*}}+\frac{1}{{r^{*}}^{2}}\frac{\partial \phi_{e}^{*}}{\partial \theta }\frac{\partial g_{1}^{*}}{\partial \theta })=\frac{{Pe}_{1}}{{r^{*}}^{2}\sin\theta }(\frac{\partial \phi_{e}^{*}}{\partial \theta }\frac{\partial \psi^{*}}{\partial r^{*}}-\frac{\partial \phi_{e}^{*}}{\partial r^{*}}\frac{\partial \psi^{*}}{\partial \theta })\;1\leq r^{*}<R_{W}^{*}$$

(36) $${\nabla^{*}}^{2}g_{2}^{*}+\alpha (\frac{\partial \phi_{e}^{*}}{\partial r^{*}}\frac{\partial g_{2}^{*}}{\partial r^{*}}+\frac{1}{{r^{*}}^{2}}\frac{\partial \phi_{e}^{*}}{\partial \theta }\frac{\partial g_{2}^{*}}{\partial \theta })=\frac{{Pe}_{2}}{{r^{*}}^{2}\sin\theta }(\frac{\partial \phi_{e}^{*}}{\partial \theta }\frac{\partial \psi^{*}}{\partial r^{*}}-\frac{\partial \phi_{e}^{*}}{\partial r^{*}}\frac{\partial \psi^{*}}{\partial \theta })\;1\leq r^{*}<R_{W}^{*}$$

(37) $$E^{4}\psi^{*}=-\frac{(\kappa a{)}^{2}}{1+\alpha }\left\{\begin{array}{c} \left[\frac{\partial g_{1}^{*}}{\partial r^{*}}\exp(-\phi_{e}^{*})+\frac{\partial g_{2}^{*}}{\partial r^{*}}\alpha \exp(\alpha \phi_{e}^{*})\right]\frac{\partial \phi_{e}^{*}}{\partial \theta }\\ -\left[\frac{\partial g_{1}^{*}}{\partial \theta }\exp(-\phi_{e}^{*})+\frac{\partial g_{2}^{*}}{\partial \theta }\alpha \exp(\alpha \phi_{e}^{*})\right]\frac{\partial \phi_{e}^{*}}{\partial r^{*}} \end{array}\right\}\sin\theta \;1\leq r^{*}<\infty$$

(38) $$E^{*4}\psi_{I}^{*}=0\;0\leq r^{*}<1$$

where the dimensionless stream function is introduced to eliminate the pressure term.

The resulting boundary conditions in dimensionless form are as follows:

(39) $${\varepsilon_{m}\left[\frac{\partial \phi_{e}^{*}}{\partial r^{*}}\right]}_{r^{*}=1^{+}}-\varepsilon_{D}{\left[\frac{\partial {\phi_{e}}_{I}^{*}}{\partial r^{*}}\right]}_{r^{*}=1^{-}}=-\sigma^{*}{\;r}^{*}=1$$

(40) $$\frac{\partial \phi_{e}^{*}}{\partial r^{*}}=-\sigma^{*}{\;r}^{*}=1$$

(41) $$\delta \phi^{*}=0{\;r}^{*}=1$$

(42) $$\frac{\partial g_{1}^{*}}{\partial r^{*}}=0{\;r}^{*}=1$$

(43) $$\frac{\partial g_{2}^{*}}{\partial r^{*}}=0{\;r}^{*}=1$$

(44) $${\left.\frac{\partial \Psi^{*}}{\partial r^{*}}\right|}_{r^{*}=1^{+}}={\left.\frac{\partial \Psi_{I}^{*}}{\partial r^{*}}\right|}_{r^{*}=1^{-}}{\;r}^{*}=1$$

(45) $${\tau^{*}}_{r\theta }^{N}{|}_{r^{*}=1^{+}}={\tau^{*}}_{r\theta I}^{N}{|}_{r^{*}=1^{-}}{\;r}^{*}=1$$

(46) $$\phi_{e}^{*}=\phi_{w}^{*}=0{\;r}^{*}=R_{w}^{*}$$

(47) $$\frac{\partial \delta \phi^{*}}{\partial R^{*}}=0{\;r}^{*}=R_{w}^{*}$$

(48) $$\frac{\partial g_{j}^{*}}{\partial R^{*}}=0{\;r}^{*}=R_{w}^{*}$$

(49) $$\psi^{*}=0{\;r}^{*}=R_{w}^{*}$$

(50) $$\frac{\partial \psi^{*}}{\partial R^{*}}=0{\;r}^{*}=R_{w}^{*}$$

(51) $$\psi^{*}=\frac{1}{2}U^{*}R^{*2}+\psi_{\infty }^{*}\left(R^{*}\right),\frac{\partial \psi^{*}}{\partial Z^{*}}=0{\;Z}^{*}=\pm L^{*}$$

(52) $$\phi_{e}^{*}=\phi_{e,\infty }^{*}\left(R^{*}\right)=0{\;Z}^{*}=\pm L^{*}$$

(53) $$\frac{\partial \delta \phi^{*}}{\partial Z^{*}}=-\beta \nabla^{*}C^{*}{\;Z}^{*}=\pm L^{*}$$

(54) $$g_{j}^{*}=\left(\beta -1\right)\;\nabla^{*}C^{*}r^{*}cos\theta {\;Z}^{*}=\pm L^{*}$$

### 4.2. Numerical Scheme: Domain Decomposition and Patched Pseudo-Spectral Method

To handle complex geometry like the cylindrical pore considered here, the physical domain is decomposed into multiple subregions first. Each and every subregion is then conformally mapped to form a corresponding rectangular computational mathematical domain. This enables the application of the pseudo-spectral method in each subdomain. Continuity of all variables and their derivatives is assumed to be maintained across each interface. This treatment is referred to as the patched pseudo-spectral method here, an extended formulation suitable for complicated domains.

**Figure 7:**
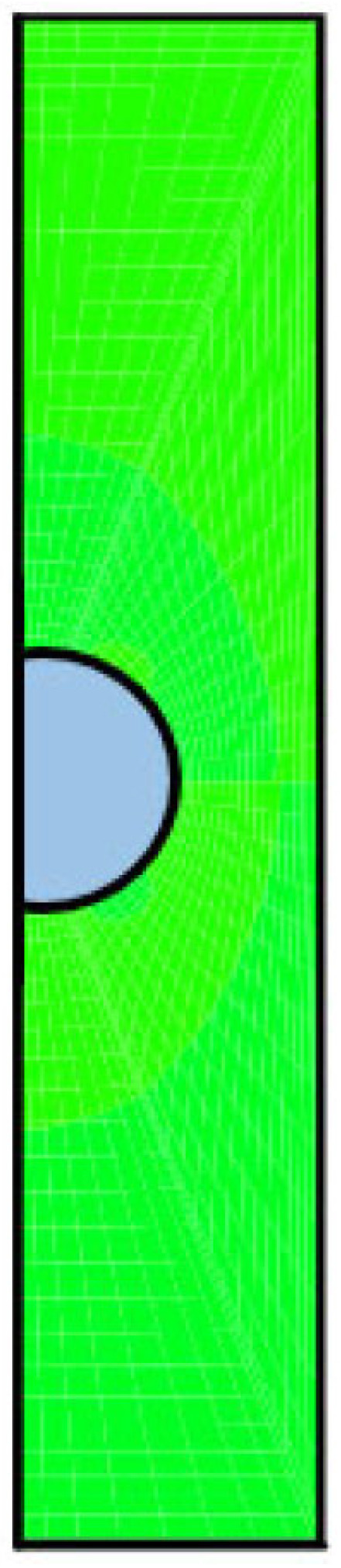
Two-dimensional physical domain, where the green colored one refers to the electrolyte solution filling the cylindrical pore, whereas the blue colored one refers to the conducting droplet. Only half of the actual physical domain is shown due to the axisymmetry of the system.

The axisymmetric nature of the problem reduces it to a two-dimensional half domain, as shown in Figure 7.

**Figure 8:**
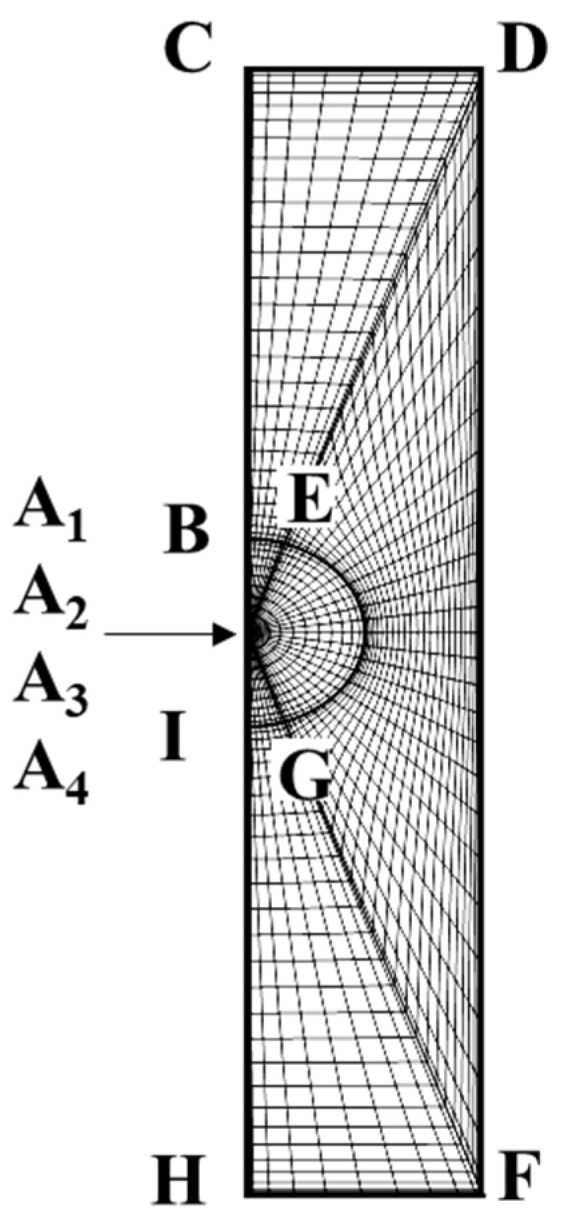
Mesh diagram of sub-regions before conformal mapping.

The pseudo-spectral method cannot be applied directly to this region though as the method requires an orthogonal geometric domain to lay out collocation points in each direction for further mathematical treatments and simplifications. Moreover, it is difficult to express in explicit form the precise location of a cylindrical pore wall and the upstream/downstream planes using spherical coordinates adopted for the droplet interior region. To reconcile with this irregular domain problem as well as the appropriate expression of the boundary location, the two-dimensional physical domain in Figure 7 is further decomposed into three separate subregions as shown in Figure 8, where the uppercase English letters indicate the locations of the geometric domain before and after the conformal mapping.

**Figure 9:**
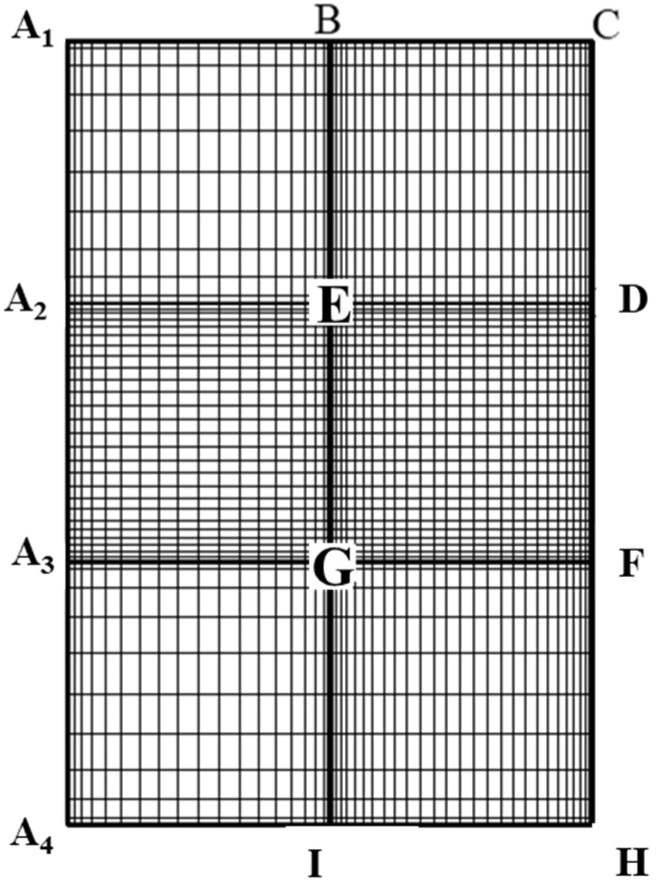
Orthogonal mesh diagram of sub-regions after conformal mapping.

The mesh is further transformed to the corresponding rectangular region (x, y) as shown in Figure 9 via a conformal mapping. The details can be found elsewhere [30].

Figure 9 is the ultimate mathematical domain for computation with the pseudo-spectral method adopted in each subdomain and continuity of each variable and its derivative is enforced at each interface of the six sub-regions shown. This type of treatment is referred to as the “patched pseudo-spectral method”, which is an extension of the original classic pseudo-spectral method. Details of this mapping can be found elsewhere [31].

The pseudo-spectral method is a member of the family referred to as the method of weighted residuals (MWRs) [32]. As for the pseudo-spectral method in general, which is also referred to as the orthogonal collocation (OC) method, the solution is approximated as a power series expansion of some orthogonal functions, for instance:

(55) $$Y_{n}(x)=\sum_{n=0}^{N}a_{n}T_{n}\left(x\right)$$

where $T_{n}\left(x\right)$ is the set of orthogonal functions selected; $a_{n}$ is the coefficients to be determined; N is the highest order of the approximating functions; and $T_{n}\left(x\right)$ is the n-th order Chebyshev polynomial. The n-th order approximating function $Y_{n}\left(x\right)$ is substituted into the original system of ordinary differential equations to generate a function referred to as the residual, R_i_ (x), which is made orthogonal to a series of weighting functions $w_{j}(x)$ as follows:

(56) $$\int_{domain}w_{j}\left(x\right)R_{i}\left(x,a_{n}\right)dx=0\;\mathrm{in}\mathrm{general}.$$

where $w_{j}(x)$ is chosen as the Delta function $\delta \left(x-x_{k}\right)$ here for the pseudo-spectral method, or the orthogonal collocation method with $x_{k}$’s referred to as the collocation points.

For the Chebyshev polynomials used here, $x_{k}$ is chosen as the j-th root of the $T_{n}\left(x\right)$, which is referred to as the “collocation point”. The coefficients $a_{n}$ thus can be evaluated via the above residual equations. Note that the above definite integral of residual equations, Equation (56), can be regarded as an extension of the “orthogonal” concept with the functions resembling vectors of infinite dimensions and the integration resembling the inner product. For Chebyshev polynomials, the roots are analytically obtainable. Moreover, the choice of the Delta function as the weighting function makes the integration very simple:

(57) $$\int_{domain}\delta \left(x-x_{k}\right)R_{i}\left(x,a_{n}\right)dx=R_{i}\left(x_{k},a_{n}\right)$$

The details can be found elsewhere.

### 4.3. Evaluation of Droplet Mobility

After solving the coupled electric and flow fields using the developed numerical algorithm, the resulting dimensionless hydrodynamic drag force (F_Dz_*) and electric driving force (F_Ez_*) acting on the droplet surface can be evaluated as follows:

(58) $$F_{Dz}^{*}=\pi \int_{0}^{\pi }{\left|r^{*4}\sin^{3}\theta \frac{\partial }{\partial r^{*}}\left(\frac{E^{*2}\psi^{*}}{r^{*2}\sin^{2}\theta }\right)\right|}_{r^{*}=1}d\theta -\;\pi \int_{0}^{\pi }{\left|r^{*2}\sin^{2}\theta \left\{\frac{(\kappa a{)}^{2}}{1+\alpha }\left[\exp(-\phi_{e}^{*})\left(1-\delta \phi^{*}-g_{1}^{*}\right)-\exp(\alpha \phi_{e}^{*})(1+\alpha (\delta \phi^{*}+\;g_{2}^{*}))\right]\frac{\partial \phi_{e}^{*}}{\partial \theta }+\left[\exp(-\phi_{e}^{*})-\exp(\alpha \phi_{e}^{*})\right]\frac{\partial \delta \phi^{*}}{\partial \theta }\right\}\right|}_{r^{*}=1}d\theta$$

(59) $$F_{Ez}^{*}=\pi \int_{0}^{\pi }{\left|\left\{r^{*2}\sin\theta \cos\theta \left[{\left(\frac{\partial \phi_{e}^{*}}{\partial r^{*}}\right)}^{2}+2\frac{\partial \phi_{e}^{*}}{\partial r^{*}}\frac{\partial \delta \phi^{*}}{\partial r^{*}}\right]+r^{*2}\sin^{2}\theta \left(\frac{\partial^{2}\phi_{e}^{*}}{\partial r^{*}\partial \theta }\frac{\partial \phi_{e}^{*}}{\partial r^{*}}+\;\frac{\partial^{2}\phi_{e}^{*}}{\partial r^{*}\partial \theta }\frac{\partial \delta \phi^{*}}{\partial r^{*}}+\frac{\partial \phi_{e}^{*}}{\partial r^{*}}\frac{\partial^{2}\delta \phi^{*}}{\partial r^{*}\partial \theta }+\frac{1}{r^{*2}}\frac{\partial \phi_{e}^{*}}{\partial \theta }\frac{\partial^{2}\phi_{e}^{*}}{\partial \theta^{2}}+\frac{1}{r^{*2}}\frac{\partial^{2}\phi_{e}^{*}}{\partial \theta^{2}}\frac{\partial \delta \phi^{*}}{\partial \theta }+\frac{1}{r^{*2}}\frac{\partial \phi_{e}^{*}}{\partial \theta }\frac{\partial^{2}\delta \phi^{*}}{\partial \theta^{2}}\right)-\;2r^{*}\sin^{2}\theta \left(\frac{\partial \phi_{e}^{*}}{\partial r^{*}}\frac{\partial \phi_{e}^{*}}{\partial \theta }+\frac{\partial \phi_{e}^{*}}{\partial r^{*}}\frac{\partial \delta \phi^{*}}{\partial \theta }+\frac{\partial \delta \phi^{*}}{\partial r^{*}}\frac{\partial \phi_{e}^{*}}{\partial \theta }\right)\right\}\right|}_{r^{*}=1}d\theta$$

The diffusiophoretic mobility of the droplet, defined as the ratio of its velocity to the magnitude of the imposed concentration gradient, is given by the following:

(60) $$\mu^{*}=\frac{U^{*}}{\nabla^{*}C^{*}}=-\frac{F_{E}^{*}}{F_{D}^{*}}$$

Following the classic approach proposed by O’Brien and White [26], the droplet mobility can be obtained via a smart approach. The detailed mathematical treatment can be found elsewhere [31].

## 5. Conclusions

In this study, the diffusiophoretic motion of a conducting liquid metal droplet (LMD) in a cylindrical pore filled with the KCl electrolyte solution is investigated theoretically. The droplet contains no electrolytes inside and has a constant, uniform surface charge density. Corresponding governing fundamental electrokinetic equations are solved with a patched pseudo-spectral method based on Chebyshev polynomials, coupled with a geometric mapping scheme to take care of the irregular geometry of the solution domain. The results are summarized as follows:

(1) A conducting LMD always moves faster than a rigid particle. The less viscous the droplet is, the faster it moves. This is consistent with the deduction from a purely hydrodynamic consideration based on the modified Stokes law for liquid droplets. The absence of Maxwell traction tangent to the droplet surface leads to this outcome.

(2) Mobility reversal is found for droplets as the cylindrical pore becomes increasingly narrower. The droplets in narrow pores move downward to the region of lower concentration of ions; whereas they move toward the region of higher concentration of ions in wider pores. This is because the buildup of the fluid in the upstream of the droplet leads to an increase in hydrodynamic pressure there, which hinders the original upward moving tendency of the droplet and pushes it back downward instead. A negative droplet mobility is thus observed as the cylindrical pore becomes narrower. The specific force balance between the electric driving force hydrodynamic drag force determines the ultimate droplet motion pattern. The narrow pore leads to a significant reduction in the area available for fluid flow and a severe deformation of the motion-inducing double layer polarization, which are the underlying electrokinetic mechanisms.

(3) “Solidification phenomenon” is observed where the droplet moves with identical speed like a rigid particle without the interior recirculating vortex flow regardless of its viscosity. The shear rate on the droplet surface is null under this condition, which makes the droplet resilient against possible damage from the exterior hydrodynamic shear stress when the droplet is in motion, a crucial requirement in drug delivery. Our findings here provide a guideline for the optimum liposome droplet size in its fabrication stage.

(4) “Stagnation phenomenon” is observed where the droplet ceases to move and clogs the cylindrical pore at its centerline. This happens regardless of its surface charge level and the pore size. It will happen simply if the droplet happens to have the specific radius as predicted by the stagnation point of κa. The screening/sheltering effect resulted from the large exterior vortex flow that encloses essentially the entire droplet surface is the major electrostatic reason behind it. The effective surface charge density reduces to nearly zero as a result. Moreover, the large recirculating exterior vortex flow traps the droplet hydrodynamically as well.

Overall, the findings in this study provide a comprehensive understanding of the droplet diffusiophoresis behavior in a cylindrical pore, which is a classic geometric scheme in various practical applications involving droplets, such as drug delivery and droplet microfluidic/nanofluidic operations.

## Data Availability

This article is a theoretical research study; hence, there is no data available.

## List of Symbols

a	radius of the droplet (m)
C	concentration of total electrolytes (1/m^3^)
C_0_	total concentration of electrolytes ions in the bulk solution at equilibrium state (1/m^3^)
C*	dimensionless concentration of total electrolytes defined as $C/C_{0}$
D_j_	diffusivity of ionic species j (m^2^/s)
e	elementary charge of an electron (1.6 × 10^−19^ coul)
F_DZ_*	dimensionless magnitude of the hydrodynamic drag force exerted on the droplet
F_EZ_*	dimensionless magnitude of the electric force exerted on the droplet
f_j_	molar flux of ionic species j (mole/m^2^/s)
g_j_	shape function representing the extra non-concentric concentration perturbation of ionic species j (volt)
g_j_*	dimensionless shape function representing the extra non-concentric concentration perturbation of ionic species j defined as $g_{j}/\phi_{0}$
k_B_	Boltzmann constant (1.38 × 10^−23^ joul/K)
n_j_	number density of ionic species j (1/m^3^)
n_j0_	bulk concentration of ionic species j (1/m^3^)
n_j_*	dimensionless number density of ionic species j defined as $n_{j}/n_{j0}$
P	hydrodynamic pressure
$P_{I}$	hydrodynamic pressure inside the droplet
Pe_j_	Peclet number of ionic species j (${Pe}_{j}=\frac{U_{0}a}{D_{j}}$) representing the reciprocal of its diffusivity in a dimensionless form
r	radius in spherical coordinates (r, θ, φ)
$r^{*}$	dimensionless radius in spherical coordinates, defined as r/a
R	radial distance of cylindrical coordinate (R, Z, φ)
R_b_	the cylindrical pore radius
R_w_*	the ratio of the cylindrical pore radius to the particle radius ($R_{b}/a$)
T	absolute temperature (K)
U	diffusiophoretic velocity of the droplet under consideration
U_0_	characteristic velocity in diffusiophoretic motion defined as $\frac{\varepsilon_{m}}{\eta_{m}a}{\left(\frac{k_{B}T}{z_{1}e}\right)}^{2}$
U*	dimensionless diffusiophoretic velocity defined as $U/U_{0}$
**v**	velocity vector
z_j_	charge valence of ion species j
Z	the height in cylindrical coordinate (R, Z, φ)
β	dimensionless diffusivity difference between the cations and the anions in a symmetric binary electrolyte solution defined as $\frac{D_{1}-D_{2}}{D_{1}+D_{2}}$
δϕ	perturbation of the electric potential (volt)
δϕ*	dimensionless perturbation of the electric potential defined as $\delta \phi /\phi_{0}$
$\varepsilon_{m}$	the electric permittivity of the ambient electrolyte solution (coul/volt/m)
$\varepsilon_{D}$	the electric permittivity of the droplet (coul/volt/m)
$\eta_{m}$	the viscosity of the ambient electrolyte solution
$\eta_{D}$	the viscosity of the droplet
θ	θ-coordinate in spherical coordinates (r, θ, φ)
κ	Debye length defined as $\left(\sqrt{\frac{\sum_{j=1}^{2}n_{j0}{\left(ez_{j}\right)}^{2}}{\varepsilon k_{B}T}}\right)$
μ	diffusiophoretic mobility of the particle defined as U∇C
μ*	dimensionless diffusiophoretic mobility of the particle defined as μ* = $\frac{\mu }{\frac{U_{0}}{\nabla^{*}C^{*}}}$
r	space charge density defined as $\sum_{j=1}^{N}z_{j}en_{j}$(coul/m^3^)
σ	surface charge density (coul/m^2^)
σ*	dimensionless surface charge density defined as $\frac{\sigma a}{\varepsilon_{m}\phi_{0}}$
σ_H_	viscosity ratio defined as $\eta_{D}/\eta_{m}$
ψ	stream function (m^3^/s)
$\psi^{*}$	dimensionless stream function defined as $\frac{\psi }{v_{0}a^{2}}$
$\psi_{\infty }^{*}$	the dimensionless stream function standing for the flow field within the cylindrical pore in the absence of the charged droplet
ϕ	electric potential (volt)
ϕ_0_	thermal potential in a binary electrolyte solution $\left(\phi_{0}=\;\frac{kT}{e}\right)$(volt)
ϕ_e_	equilibrium electrical potential (volt)
ϕ_e_*	dimensionless equilibrium electrical potential defined as $\phi_{e}/\phi_{0}$
ϕ_r_	dimensionless surface potential of the droplet defined as $\left(\zeta /\phi_{0}\right)$
ϕ_w_*	dimensionless electrical potential of the wall
$\tau_{r\theta }^{N}\;\;\;\;\;\;$	the hydrodynamic stress tensor
$\tau_{r\theta }^{M}\;\;\;\;\;\;$	the electric Maxwell stress tensor
ζ	electric potential on the droplet surface, or the zeta potential when the droplet is in motion (volt)

## Operators

E^2^	operator defined $E^{2}=\frac{\partial^{2}}{\partial r^{2}}+\frac{sin\theta }{r^{2}}\frac{\partial }{\partial \theta }\left(\frac{1}{sin\theta }\frac{\partial }{\partial \theta }\right)$ in spherical coordinates (r, θ, φ)
E^4^	$E^{4}=E^{2}\cdot E^{2}$
∇	gradient operator
$\nabla^{2}$	Laplacian operator
∇·	divergence operator

## Superscripts

*	dimensionless variable

## Subscripts

1	cation
2	anion
e	equilibrium state
I	interior fluid
j	j-th ionic species
∞	value at infinity

## Boldface

Boldface of variables indicates vector or tensor quantities.
